# Nutritional status and concomitant factors of stunting among pre-school children in Malda, India: A micro-level study using a multilevel approach

**DOI:** 10.1186/s12889-021-11704-w

**Published:** 2021-09-16

**Authors:** Rayhan Sk, Anuradha Banerjee, Md Juel Rana

**Affiliations:** 1 Centre for the Study of Regional Development, School of Social Sciences, JNU, New Delhi, India; 2 International Institute for Population Sciences, Mumbai, India

**Keywords:** Stunting, Low birth weight, Bidi worker, Integrated child development services, POSHAN Abhiyan, Multilevel analysis, Malda, India

## Abstract

**Background:**

Malnutrition was the main cause of death among children below 5 years in every state of India in 2017. Despite several flagship programmes and schemes implemented by the Government of India, the latest edition of the Global Nutrition Report 2018 addressed that India tops in the number of stunted children, which is a matter of concern. Thus, a micro-level study was designed to know the level of nutritional status and to study this by various disaggregate levels, as well as to examine the risk factors of stunting among pre-school children aged 36–59 months in Malda.

**Method:**

A primary cross-sectional quantitative survey was conducted using structured questionnaires following a multi-stage, stratified simple random sampling procedure in 2018. A sum of 731 mothers with at least one eligible child aged 36–59 months were the study participants. Anthropometric measures of children were collected following the WHO child growth standard. Children were classified as stunted, wasted, and underweight if their HAZ, WHZ, and WAZ scores, respectively, were less than −2SD. The random intercept multilevel logistic regression model has been employed to estimate the effects of possible risk factors on childhood stunting.

**Results:**

The prevalence of stunting in the study area is 40% among children aged 36–59 months, which is a very high prevalence as per the WHO’s cut-off values (≥40%) for public health significance. Results of the multilevel analysis revealed that preceding birth interval, low birth weight, duration of breastfeeding, mother’s age at birth, mother’s education, and occupation are the associated risk factors of stunting. Among them, low birth weight (OR 2.22, 95% CI: 1.44–3.41) and bidi worker as mothers’ occupation (OR 1.92, 95% CI: 1.18–3.12) are the most influencing factors of stunting. Further, about 14 and 86% variation in stunting lie at community and child/household level, respectively.

**Conclusion:**

Special attention needs to be placed on the modifiable risk factors of childhood stunting. Policy interventions should direct community health workers to encourage women as well as their male partners to increase birth interval using various family planning practices, provide extra care for low birth weight baby, that can help to reduce childhood stunting.

**Supplementary Information:**

The online version contains supplementary material available at 10.1186/s12889-021-11704-w.

## Background

The latest edition of the Global Nutrition Report 2018 addressed that India tops in the number of stunted children (46.6 million), followed by Nigeria (13.9 million) and Pakistan (10.7 million). These three countries are home to almost half of the world’s stunted (47.2%) children [1]. A recent study published in Lancet found that malnutrition was the main cause of death among children below 5 years in every Indian state in 2017, accounting for 68.2% of the total deaths among children below 5 years of age [2]. The latest round of the Indian family and health survey shows that about 38, 21, and 36% of children are stunted, wasted and underweight, respectively, at the national level in 2015–16 [3]. While in Malda district, about 38, 23, and 37% of children aged under 5 years are stunted, wasted and underweight, respectively [3].

The Government of India has tried to combat the perseverance of malnutrition through several large-scale programmes and schemes which could not reach the level of expectations mostly. Integrated Child Development Services (ICDS) is one of the most inclusive programmes for addressing child malnutrition and child well-being and development, implemented in 1975 by the Ministry of Women and Child Development, Government of India and supported by the UNICEF [4]. ICDS is operating through Anganwadi Centres (AWCs). An AWC is operated by an Anganwadi Worker (AWW) and an Anganwadi Helper (AWH). Brief detail on the ICDS is provided in the supplementary file. Recently, another flagship programme is known as National Nutrition Mission (NNM), also known as POSHAN (Prime Minister’s Overarching Scheme for Holistic Nutrition) Abhiyaan has been implemented by the Indian Government in 2017. It targets to reduce stunting by two percentages annually, under-nutrition by two percentages annually, anaemia by two percentages annually among young children, adolescent girls, and women, and reduction of low birth weight by two percentages annually. Further, it sets the target ‘Mission 25 by 2022’ for stunting to reduce it from 38.4 to 25% by 2022 in India [5]. On the other hand, sustainable development goals (SDG) recommended the target to reduce stunting is a reduction by 50% by 2030 [6].

The prevalence of malnutrition varies substantially across the states as well as by the districts of the country [3]. There are several studies on the nutritional status of children in India, especially at the national and state levels. However, the in-depth research on nutritional status, particularly among pre-school children at the micro-level, is little known. Besides these, India is a vast country, having a great diversity in its geography, religion, caste, culture, food habits, and climate. Therefore, inferences based on a particular micro-level study setting may not be applicable to another particular micro-level area for policy interventions. In this regard, a review study based in India has recommended that the risk factors of malnutrition among children should be analysed by controlling diverse measures in a given set-up [7]. In addition to that, a recent study by Menon et al. (2018) demonstrated that inter-district variations in stunting among children under 5 years old in India are largely described by a large number of health, hygiene, economic and demographic factors [8]. Based on these observations, the present study attempts to know the level of nutritional status and to study this by various disaggregate levels, as well as to examine the risk factors of stunting among pre-school children aged 36–59 months in Malda which is a district of West Bengal in India. The findings of this study can be helpful for the local governing body to adopt proper interventions for reducing the prevalence of stunting and to reach the target ‘Mission 25 by 2022’ and also the target for stunting as embodied in SDGs.

In the previous studies, it has been found that individual child characteristics, e.g., child’s age, sex, birth interval, birth order, and birth weight, are the associated factors of stunting [9–14]. Many studies found that children born with low birth weight are more prone to become stunted in their childhood [15–21]. A multi-country analysis based in 137 developing countries found that preterm birth or short gestational age is the leading cause of stunting among children of 24–35 months old [22]. Investigations revealed that birth interval is also another leading risk factor of stunting among children in developing countries, where along with the increase in the birth interval, the probability of stunting decreases [23–26]. In the Indian context, researchers have also demonstrated that birth interval is an important factor of stunting among children [27–31]. A cohort study based in Kenya exhibited that a longer duration of breastfeeding is positively associated with child growth [32]. Further, it has been found that mothers’ characteristics, e.g., maternal age at birth, mother’s education, maternal nutritional and occupational status affect stunting among children. A study based in India showed that maternal age is positively associated with stunting among children [28], while some studies showed that stunting is inversely related to maternal age [33, 34]. Many studies have documented that with the increase of maternal education, the chances of stunting decrease among children [14, 24, 35–38]. Studies also reported that mothers’ employment is related to poor nutritional status among children [39]. On the contrary, a study found that the employment status of mothers does not matter for stunting, but the type of remuneration does matter for the same, in which children of unpaid workers are significantly more stunted than the paid workers [40]. It has also been observed that household characteristics, e.g. religion, caste or social groups [14, 35], wealth index, and the place of residence [41, 42], are also associated factors of stunting among children. Besides these, studies that evaluated the effects of ICDS at the national level in India show that there are no significant effects on reducing stunting among children who are accessing the services from ICDS [43–45].

## Methods

### Study design, setting, sample size

A primary cross-sectional quantitative survey was conducted in the Malda district of West Bengal, India, in 2018 (a brief description of Malda is provided in the supplementary file). This included a structured questionnaire consisting of children’s anthropometric measures, child demographic characteristics, maternal socio-demographic characteristics, and household characteristics that have been used to collect the information. In the beginning, the questionnaire was prepared in the English language, and thereafter it was translated into Bengali, which is the local language of the study area. The questionnaire used in this survey was developed for the PhD project only, and the present study is a part of it (see the questionnaire in supplementary file). The data were collected from the rural and urban areas from the 12 selected PSUs (primary sampling units), which were nested within the three selected blocks and a municipality in the Malda district. The villages in rural areas and wards in urban areas were considered as the PSUs. In general, sub-districts, i.e., Tehsils or Taluks or Blocks, are the administrative units under a district in rural India. On the other hand, a municipality is an urban local body in India. Considering the researcher’s time, affordability, and feasibility, a sum of 750 samples were expected to be collected.

### Participants

Mothers with at least one pre-school-aged child (36–59 months old) living in the households were the study participants in this research.

### Sample selection procedure

A multi-stage, stratified simple random sampling procedure was used to collect the survey samples. The statistics from the Census of India 2011 were used for framing the sampling procedure. In the beginning, the district population was stratified by the rural and urban population, and the samples were distributed according to the proportion to population size. According to the Census 2011, 13.6 and 86.4% of the population were urban and rural residents, respectively. Thus, the total expected sample for the urban and rural population was 102 (750/100*13.6) and 648 (570/100*86.4), respectively.

There were 15 Blocks or Tehsils, the higher-level administrative units in rural areas in the Malda district. These were stratified into three groups using the tertile method after ranking them by z-scores of relative wealth index that was a composite measure, constructed with certain variables like household’s assets, amenities, and facilities using data from the Census of India, 2011 [46]. Afterward, one block had been selected randomly from each group in the first stage of sampling. The tertile method for stratification was used to represent the children from all the sections of the Malda district. A total of three blocks had been selected, and the sample size had been distributed according to the proportion to population size method. On the other hand, in the case of urban areas, a municipality had been selected randomly from the two municipalities, and all the urban samples had been taken from that selected municipality.

In the second stage of sampling, PSUs or villages/wards had been selected. Likewise blocks stratification, villages/wards were also stratified into three groups following the same process. After this, one village/ward had been selected randomly from each group of villages/wards. Thus, three PSUs had been selected from each selected block or municipality. A sum of 12 PSUs; nine PSUs (villages) from rural areas, and three PSUs (wards) from urban areas had been selected. Here, at the village/ward level, the samples had been fixed equally by dividing the total expected sample by the total selected villages/wards for the corresponding block or municipality. Further, there were Anganwadi Centres (AWCs) in each selected village or wards, which were not involved in the sampling procedure, although a question was asked to the respondents as to which AWC is being accessed by children or listed as beneficiary holders for measuring the variation in outcome interest between the AWC centres. A sum of 42 AWCs was nested within 12 selected villages or wards. Almost all the children were listed in the AWC’s record file for a designated area under an AWC. Here, in this study, AWCs were considered as communities.

Since the present research aims to study the nutritional status and childhood development among children aged 36 to 59 months, therefore, only households that had at least one child of age ranging between 36 to 59 months were the study participants of the present study. Thus, the listing of households with at least an eligible child had been made from various sources in the selected PSUs. In the final stage, after completion of house listing, the desired number of households for the corresponding PSU had been selected using a simple random sampling procedure. After selecting the households with at least one child aged between 36 and 59 months for the survey, two major conditions had been followed for selecting a child with a mother or caregiver who was the primary respondent of the survey questionnaire. These were, (i) child must be between 36 to 59 months of old at the time of the survey; (ii) if there were more than one child aged between 36 to 59 months in a selected household, in that case, only one child had been selected following the alphabetic order of child’s name, and the survey questions had been asked to the mother or caregiver for that particular selected child. The expected sample size was 750. However, a sum of 731 mothers or caregivers had responded and completed the survey, and the remaining 19 samples were not interviewed due to either unavailability of the respondents or their non-response to the survey. Thus, the overall survey response rate was 97.5%. Regarding fieldwork, see the supplementary file.

### Outcome variable

Anthropometric measures of children such as age, height (standing), and weight (standing) were collected following the WHO child growth standards [47]. Children’s anthropometric data were transferred to the WHO Anthro software version 3.2.2, 2011 [48], and the Z-scores of the index for height-for-age Z-score (HAZ), weight-for-height Z-score (WHZ), and weight-for-age Z-score (WAZ) were computed using WHO Child Growth Standards, 2006. Children were classified as stunted, wasted, and underweight if their HAZ, WHZ, and WAZ scores, respectively, were less than −2SD of the WHO Child Growth Standards median following the WHO guidelines [49]. A binary variable has been created for all these nutritional outcomes using STATA version 13.1 [50]. However, the main outcome variable in this study was stunting because it is a chronic form of under-nutrition.

### Explanatory variables

A sum of 14 potential explanatory variables was chosen to examine the risk factors of stunting based on the findings of previous studies and the availability of the data in the present study. Among child’s characteristics, age of the child (categorical: 36–47 months, 48–59 months), sex of the child (categorical: male, female), birth order (categorical: 1, 2, 3, ≥4), low birth weight (dummy: no vs yes), preceding birth interval (categorical: < 36 months, ≥36 months, first birth) and duration of breastfeeding (categorical: ≤12 months, 13–24 months, 25–36 months, ≥37 months) were selected. Low birth weight is defined by the World Health Organization as the weight of lower than 2500 g or 5.5 pounds at the time of birth [51]. Mother’s age at birth, birth history, last menstrual period (LMP) for a selected child, and date of birth, as well as birth weight of a selected child, were collected from the mother/child immunisation card. Information about LMP and birth weight were missing for almost 20% (*n* = 146) and 6% (*n* = 46) cases in the study sample, respectively. Also, they are correlated with each other. Thus, a dummy variable was created for low birth weight of below 2500 g referred to as ‘yes’ and ‘no’ for other than that. Maternal age at birth (categorical: < 20 years, 20–25 years, > 25 years), mother’s education (categorical: none, primary, lower secondary, upper secondary, higher), mother’s occupation [categorical: housewife/housemaker, agricultural worker or manual labourer, bidi worker, others (public/private services, other professional workers)] were taken from mother’s characteristics. Bidi, also spelt as beedi or biri, is a popular local mini-cigar or cigarette in India. It is filled with tobacco flake and wrapped by tendu leaf, made by women mostly in their homes. Bidi making is one of the main sources of livelihood in rural areas in the study region. Among household characteristics, religion [categorical: Hindu, Muslim or others (minor religious groups viz. Christian and Bidwin)], social groups or caste [categorical: general, OBC (Other Backward Class), SC (Scheduled Caste), ST (Scheduled Tribe)], wealth index (Categorical: poor, middle, rich) and the place of residence (categorical: urban, rural) were chosen. The wealth index was used as a proxy measure of income that has been constructed using the selected household’s amenities, facilities, and assets following the Demographic Health Survey (DHS) [52]. Children accessing or attending the AWCs of ICDS schemes (categorical: no, yes) was a service factor taken from the malnutrition eradication programme. The first category of each of the above-described variables was considered as a reference group in the multivariate regression analysis.

### Ethics

Ethical approval was obtained from the IERB-JNU (Institutional Ethics Review Board- Jawaharlal Nehru University), New Delhi, IERB Ref. No.2019/Student/223. The purpose of the study was thoroughly explained to respondents, and written consents were obtained from the parents/guardians of the minors included (minors are considered anyone under the age of 16) before starting the survey. Also, the written consent information for all mothers and caregivers who participated in the study was obtained before starting the survey.

### Statistical analysis

Univariate descriptive statistics were carried out to describe the sample characteristics and to explore the pattern of the outcome variable. Since the dataset for the present study was hierarchically stratified, 731 children are nested within the 731 mothers/caregivers, mothers are nested within 731 households, and the households are nested within 42 Anganwadi Centres (AWCs) of the ICDS scheme. Thus, bearing in mind this hierarchical structure of the dataset, a random intercept multilevel logistic regression model was employed to estimate the effects of possible risk factors on childhood stunting. Also, multilevel modelling was used to estimate the variation in stunting at the lowest level of hierarchy to a higher one, is the advantaged over single-level model or simple logistic regression [42, 53]. Here, in this study, child-level or household-level and AWC level variations in stunting have been estimated. Since children are expected to have the same parental and household characteristics in their respective households, and also children belonging to a particular community have homogeneous community-level characteristics; thus it can be said that unobserved heterogeneity in outcome measure is also correlated at the household or community level [54, 55]. Therefore, a two-level logistic regression approach was employed. Level one is at the individual child level or household level, and level two is at the AWC level or community level. As only one child from a particular household had been sampled for this study, 731 selected children belong to exactly 731 households; thus, it does not make sense to consider a separate level for child and household, respectively (please see the supplementary file for model specification).

A series of the models was employed in the multilevel modelling to look at the dynamics of effects of various possible risk factors of stunting among pre-school children, e.g., child, mother, and household-level characteristics to community-level characteristics, which were entered step by step in the models. The first model (Model 1) was a null model or unconditional model where no independent variables were included. In Model 2, the child’s characteristics were included, and in the later model (Model 3), the mother’s characteristics were added, while in Model 4, only ‘child attending AWC’ was added. In Model 5, household characteristics were incorporated, while in the subsequent model, Model 6 was a complete model, place of residence was included.

## Results

Table 1 presents the univariate descriptive statistics of 731 sampled children. About more than half of the study children are male. Around one-fifth of all children were born with < 2.5 kg weight at the time of birth, defined as low birth weight. About 61% of children breastfed the WHO recommended duration of 24 months and above [56]. Among all mothers or caregivers, homemaker or housewife is the dominant category of occupation. Nearly two-thirds of children are attending AWCs or receiving supplementary food from ICDS schemes. More than half of the children belong to Muslim households. Among caste or social groups, OBC is the most prevalent caste in the study area, followed by the general category, SC and ST. About 86% of children belong to rural areas.

**Table 1:** Descriptive statistics of study children (36–59 months) and percentage distribution of stunting by background characteristics in Malda district, 2018

Characteristics	Sample (%)	Sample (number)	Stunting (%)
**Child’s age**			
36–47 Months	47.7	349	40.7
48–59 Months	52.3	382	39.5
**Child’s sex**			
Male	51.6	377	37.4
Female	48.4	354	42.9
**Birth order**			
1	41.7	305	35.7
2	34.9	255	38.0
3	13.1	96	52.1
≥ 4	10.3	75	49.3
**Birth interval**			
< 36 Months	23.1	169	52.1
≥ 36 Months	33.0	241	37.3
First birth	43.9	321	35.8
**Low birth weight**			
No	80.6	589	37.0
Yes	19.4	142	52.8
**Duration of breastfeeding**			
≤ 12 Months	08.3	61	50.8
13–24 Months	31.1	227	45.4
25–36 Months	48.3	353	37.1
≥ 37 Months	12.3	90	31.1
**Mother’s age at birth**			
< 20 Years	19.3	141	33.3
20–25 Years	49.1	359	39.3
> 25 Years	31.6	231	45.5
**Mother’s education**			
None	18.3	134	55.2
Primary	15.7	115	53.9
Lower secondary	35.6	260	35.8
Upper secondary	20.1	147	36.1
Higher	10.3	75	14.7
**Mother’s occupation**			
Homemaker	59.8	437	33.9
Agricultural/manual worker	08.1	59	66.1
Bidi worker	27.8	203	49.3
Other	04.4	32	18.8
**Child attending AWC**			
No	39.3	287	28.9
Yes	60.7	444	47.3
**Religion**			
Hindu	43.9	321	31.8
Muslim/Others	56.1	410	46.6
**Caste**			
General	31.5	230	30.4
OBC	42.8	313	44.4
SC	18.1	132	34.1
ST	07.7	56	69.6
**Wealth index**			
Poor	29.3	214	52.8
Middle	37.4	273	42.9
Rich	33.4	244	25.8
**Place of residence**			
Urban	13.7	100	19.0
Rural	86.3	631	43.4
**All**	**100.0**	**731**	**40.1**

Source: Computed from the primary field survey, Malda, 2018

**Fig. 1:**
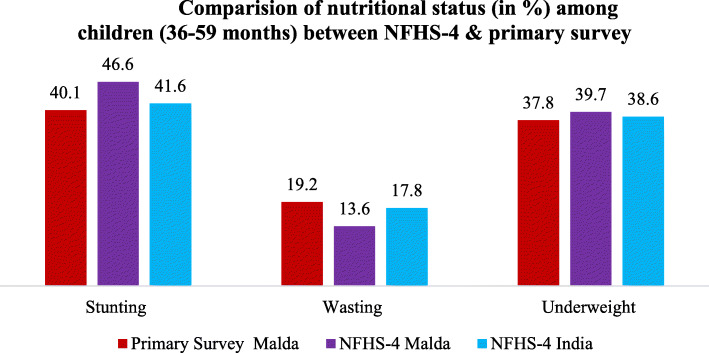
Comparision of nutritional status (in %) among children (36–59 months) between NFHS-4 & primary survey

Figure 1 depicts a comparison between NFHS-4 and primary survey estimation of nutritional status among children of 36–59 months old. Estimation from the primary field survey data shows that about 40, 19, and 38% of children aged 36–59 months are stunted, wasted and underweight, respectively, in Malda. While the estimation from the NFHS-4 demonstrates that the prevalence of stunting, wasting, and underweight are about six percentage points higher, six percentage points lower and two percentage points higher, respectively, in Malda than the estimation from the primary data of Malda. However, the estimation from NFHS-4 for India shows that the prevalence of stunting, wasting, and underweight are almost equal to the estimation from primary survey data for Malda district.

The bivariate estimations of childhood stunting by background characteristics of children in the Malda district are also presented in Table 1. Female children are almost six percentages points more stunted than male children. By and large, the prevalence of stunting increases from the first birth order to the higher order of birth. Children who born with < 36 months of the preceding birth interval are about 15% points more stunted than those born with ≥36 months preceding birth spacing. Similarly, children who born with < 2.5 kg weight are 16% points more stunted than those born with 2.5+ kg weight. The prevalence of stunting decreases with the increasing duration of breastfeeding. With the increase in mothers’ educational level, the proportion of stunted children decreases. The prevalence of stunting among children of mothers of agricultural workers or manual labourers is the higher one in occupation factor. Unexpectedly, children who are accessing the AWCs almost 18% points more stunted as compared to those who do not access the same. The prevalence of childhood stunting is highest among the ST population in comparison to other groups. Children belonging to Hindu households are about 15% points less stunted than children of Muslim or other households. Children from rural areas are almost 25% points more stunted as compared to the children living in urban areas.

Table 2 presents the results of multivariate multilevel logistic regression showing the effects of fixed-effects parameters as well as random-effects parameters, e.g., individual child and community-level effects on stunting among children in the collected sample. A lower Wald chi-square statistic (< 0.001) for all the conditional models implies that childhood stunting varied significantly by the individual, mother, household, and community-level factors. In the random-effect part, the *p*-values of the log-likelihood ratio test (LR) versus logistic regression vary from 0.001 to 0.024, which also indicate that the variance in child stunting differs significantly by an individual child or household level and AWC or community level.

**Table 2:** Estimated adjusted odds ratio [95% Confidence Intervals] using multilevel logistic regression for stunting among children (36–59 months) in Malda district, 2018

Characteristics	Model 1	Model 2	Model 3	Model 4	Model 5	Model 6
***Fixed effects***						
**Child’s age**						
36–47 Months®						
48–59 Months		0.96 [0.69–1.33]	1.00 [0.72–1.41]	1.07 [0.76–1.51]	1.05 [0.75–1.48]	1.05 [0.75–1.48]
**Child’s sex**						
Male®						
Female		1.29 [0.93–1.78]	1.20 [0.86–1.68]	1.21 [0.87–1.70]	1.22 [0.87–1.70]	1.23 [0.88–1.73]
**Birth order**						
1®						
2		0.68 [0.25–1.88]	0.64 [0.23–1.80]	0.63 [0.23–1.77]	0.61 [0.22–1.71]	0.60 [0.22–1.69]
3		1.04 [0.34–3.14]	0.72 [0.23–2.22]	0.70 [0.22–2.15]	0.61 [0.20–1.91]	0.60 [0.19–1.86]
≥ 4		0.83 [0.26–2.66]	0.53 [0.16–1.78]	0.52 [0.16–1.75]	0.46 [0.14–1.55]	0.46 [0.14–1.53]
**Birth interval**						
< 36 Months®						
≥ 36 Months		0.62*[0.40–0.96]	0.57*[0.36–0.89]	0.58*[0.37–0.91]	0.60*[0.38–0.94]	0.59*[0.38–0.94]
First birth		0.42 [0.15–1.20]	0.51 [0.18–1.45]	0.52 [0.18–1.49]	0.53 [0.18–1.53]	0.53 [0.18–1.52]
**Low birth weight**						
No®						
Yes		2.28***[1.50–3.46]	2.31***[1.50–3.55]	2.21***[1.44–3.41]	2.23***[1.45–3.43]	2.22***[1.44–3.41]
**Duration of breastfeeding**						
≤ 12 Months®						
13–24 Months		0.75 [0.40–1.42]	0.74 [0.38–1.42]	0.75 [0.39–1.44]	0.71 [0.37–1.37]	0.69 [0.36–1.34]
25–36 Months		0.56 [0.30–1.02]	0.55 [0.30–1.03]	0.55 [0.29–1.02]	0.55 [0.29–1.02]	0.53*[0.28–1.01]
≥ 37 Months		0.38*[0.18–0.80]	0.33**[0.15–0.71]	0.34**[0.15–0.74]	0.37*[0.17–0.81]	0.37*[0.17–0.81]
**Mother’s age at birth**						
< 20 Years®						
20–25 Years			1.38 [0.84–2.25]	1.43 [0.87–2.34]	1.56 [0.95–2.57]	1.59 [0.97–2.61]
> 25 Years			1.61 [0.86–3.04]	1.66 [0.88–3.13]	1.90*[1.00–3.62]	1.98*[1.04–3.78]
**Mother’s education**						
None®						
Primary			1.42 [0.79–2.55]	1.44 [0.80–2.59]	1.48 [0.82–2.68]	1.51 [0.83–2.73]
Lower secondary			0.70 [0.41–1.21]	0.73 [0.43–1.27]	0.82 [0.47–1.43]	0.82 [0.47–1.44]
Upper secondary			0.95 [0.50–1.82]	1.02 [0.53–1.96]	1.16 [0.59–2.27]	1.20 [0.61–2.36]
Higher			0.26**[0.11–0.62]	0.29**[0.12–0.71]	0.35*[0.14–0.89]	0.36*[0.14–0.92]
**Mother’s occupation**						
Housemaker®						
Agricultural/manual worker			3.14**[1.41–7.00]	2.82*[1.26–6.28]	1.84 [0.58–5.86]	1.77 [0.55–5.66]
Bidi worker			2.10***[1.29–3.40]	1.98**[1.22–3.21]	2.03**[1.25–3.29]	1.92**[1.18–3.12]
Other			0.85 [0.30–2.36]	0.79 [0.28–2.22]	0.88 [0.32–2.45]	0.85 [0.30–2.36]
**Child attending AWC**						
No®						
Yes				1.54*[1.04–2.28]	1.40 [0.95–2.08]	1.40 [0.95–2.08]
**Religion**						
Hindu®						
Muslim/others					1.48 [0.85–2.56]	1.47 [0.85–2.54]
**Caste**						
General®						
OBC					1.11 [0.62–1.96]	1.00 [0.56–1.79]
SC					1.02 [0.55–1.91]	0.96 [0.51–1.81]
ST					1.96 [0.53–7.32]	1.87 [0.50–6.98]
**Wealth index**						
Poor®						
Middle					1.00 [0.65–1.53]	1.00 [0.65–1.54]
Rich					0.74 [0.43–1.28]	0.83 [0.47–1.46]
**Place of residence**						
Urban®						
Rural						1.72 [0.78–3.80]
***Random effects***						
**Random Effects Variance (SE)**						
Community level	0.73 (0.14)	0.69 (0.15)	0.54 (0.14)	0.52 (0.14)	0.42 (0.15)	0.52 (0.14)
**ICC**						
Community level	0.18	0.17	0.14	0.14	0.11	0.14
**Variance decomposition** (percentage by level)						
Child level/household level	81.85	82.64	86.00	86.30	88.71	86.30
Community/AWC level	18.15	17.36	14.00	13.70	11.29	13.70
**Model fit statistics**						
Wald test X2		33.74	69.13	72.75	80.67	81.96
Probability >chi-square		0.000	0.000	0.000	0.000	0.000
LR test vs. logistic regression: *p-*value	0.000	0.000	0.001	0.001	0.016	0.024

*ICC* Intra-class correlation coefficient, *SE* Standard error, *LR* likelihood ratio, *®* Reference category; *** significant level at *p*-values = < 0.001, ** significant level at *p*-values = < 0.010, and * significant level at *p*-values = < 0.050

Source: Computed from the primary field survey, Malda, 2018

Model 6 is a final and comprehensive model. After controlling for all the explanatory variables in this model, it is found that birth interval, low birth weight, duration of breastfeeding, mother’s age at birth, mother’s education, and occupation are the significant risk factors of stunting. Among them, low birth weight (OR 2.22, 95% CI: 1.44–3.41) and bidi worker (OR 1.92, 95% CI: 1.18–3.12) are the most influencing factors of stunting, which show a consistent effect throughout the models. Besides these, the mother’s age of > 25 years at birth (OR 1.98, 95% CI: 1.04–3.78) is also another important risk factor of stunting. Nevertheless, the proportion of stunted children among the mothers who are agricultural workers or manual labourers and children belonging to the ST community are extremely high, as shown by bivariate estimations, but these are not significant risk factors of stunting in the full regression model. On the other hand, the value of the community level ICC is 0.14, which indicates the correlation among the children within the same community is 0.14 in the case of child stunting. In other words, about 14% variation in stunting lies at AWC or community level, and the larger part of the variation in stunting lies at the child or household level, as shown in the random part of the model. Further, the highest degree of changes in the random part of the model is observed after including the mother’s characteristics in the third model.

## Discussion

The prevalence of stunting in the study area is 40% among pre-school children aged 36–59 months, which is a very high prevalence as per the WHO’s cut-off values (≥40%) for public health significance [49]. If the WHO’s cut-off value (≥40%) is considered as the very high level of prevalence, several categories of background characteristics of children have shown a very high prevalence of stunting in Malda district, e.g., female child, 3+ birth order, < 36 birth intervals, low birth weight, < 25 months of duration of breastfeeding, > 25 years of mother’s age at birth, illiterate or poorly educated mothers, mothers’ occupation as agricultural workers or manual labourers and bidi workers, children attending AWCs, children belong to Muslim or other minor religious groups, children belong to OBC and ST categories, children living in poor as well as middle-class family, and children living in rural areas. However, the highest proportion of stunted children is found among the most deprived ST community, almost seven children out of 10 children are stunted in this community; followed by illiterate mothers, mothers’ occupation as agricultural workers or manual labourers, low birth weight, and poor households, where more than half of children are stunted. Here, it should be noted that after having several flagship programmes and schemes implemented by the Indian Government to eradicate malnutrition among children, still, this region, as well as India, have a very high level of prevalence of stunted children.

According to Arjan de Wagt, is the nutrition chief, UNICEF India, and his colleagues, 2019, huge potential interventions are still in the operations under the nutrition and health schemes and programmes, but the services of these programmes and schemes are not reaching the children and women at a satisfactory level. Therefore, implementation constrictions need to be appropriately addressed by that high-impact nutritional interventions can reach the maximum level, and that should be followed with the Coverage, Continuity, Intensity, and Quality (C2IQ) principle [57] in the study area as well as in India. Unexpectedly, this study found that the proportion of stunting is higher (47.3%) among the children who access the services from the AWCs or ICDS (one of the most inclusive programmes for addressing child under-nutrition) than those who are not accessing the same. Although, it might be justified by saying that children of socio-economically disadvantaged families are mostly the beneficiaries of AWCs or ICDS schemes.

The results of the multilevel analysis revealed that preceding birth interval, low birth weight, duration of breastfeeding, mother’s age at birth, mother’s education, and occupation are the only associated risk factors of stunting in this study. Low birth weight is the strongest predictor of stunting, followed by mothers’ occupation and mothers’ age at birth. Association between low birth weight and stunting has been shown by several studies [15, 18, 19, 21]. As per medical concerns, intra-uterine growth retardation (IUGR) is the main cause of low birth weight in developing countries [58]. Children who suffer from IUGR during mothers’ pregnancy are effectually born malnourished. Maternal malnutrition, low maternal weight, low maternal stature during conception, and insufficient maternal weight gain during pregnancy all together are attributed to almost half of the cases of IUGR in developing countries [20, 58]. Further, anaemia and iron deficit also accompany IUGR and subsequently results in low birth weight [59, 60]. Children of mothers engaged in bidi making are at higher risk of child stunting that requires further investigation. Usually, most of the bidi workers live in poverty, mostly below the poverty line that might be the underlying cause of the higher risk of child stunting among them. In addition to that, normally bidi making women are less careful towards their children while they sit and make the bidis; perhaps this is another reason for the higher risk of child stunting among them.

Mother’s age at birth is also another important risk factor of stunting, where children of higher maternal age are more likely to be stunted than their counterparts. A similar association is observed in another study based in India [28]. Duration of breastfeeding is also another important risk factor of stunting in this research. Children with a lower duration of breastfeeding are more likely to be stunted as compared to children with a higher duration of breastfeeding. This finding is also consistent with other studies [32, 61]. Children with lower preceding birth intervals also have higher chances of being stunted as compared to those of higher birth intervals. A similar observation is reported by other studies as well [23–25]. A birth interval may affect under-nutrition among children by its relationship with premature birth and low weight at birth. If a woman becomes pregnant too soon after birth, the mother does not get adequate time to fill their depleted body fats and nutrients during the pregnancy and breastfeeding, which may lead to premature birth and low weight at birth. Thus, a higher birth interval allows the mothers to recover and become healthy for subsequent pregnancy. Mothers with a higher birth interval can provide their children with the required nutrition for growth and a resilient immune system that reduces the chances of childhood stunting [62]. Although the effect of the ICDS schemes operates through AWCs is not a significant factor of stunting after controlling for household’s characteristics and place of residence, it does have a positive association with stunting if children are accessing the services in the case of child’s, and mother’s characteristics are only taking into consideration in the regression analysis. Likewise, the same observation is noticed in the case of the mother’s occupation, especially for agricultural workers or manual labourers. Conceivably, the multicollinearity between mother’s occupation, children accessing the services from ICDS, household and community characteristics viz. religion, caste groups, wealth status, and place of residence, is the reason for altering the effect of mother’s occupation and ICDS schemes. Therefore, further studies are needed to investigate the risk factors of stunting within the community level as well. Besides these, a higher level of changes in the level variances of the multilevel model (two-levels) was reflected after adjusting for mother’s characteristics along with the child’s characteristics. Thus, it can be said that a mother’s characteristics also play a crucial role in her child’s physical growth. On the other hand, among total variation of the two-levels model in child stunting, about 14% variation was due to the heterogeneity between the communities or AWCs. So, it can be believed that a community or particular AWC itself also has an influence on stunting among children in the study area.

## Conclusion

An attempt has been made in the present research to explore the level of nutritional status, to study this by disaggregate levels, and to examine the risk factors of stunting among pre-school children aged 36–59 months in Malda. Accordingly, a primary field survey was conducted to study the same in 2018. It was found that Malda district suffers from the burden of a high-level prevalence of child stunting. This district has already got priority from the policy-making body, and the Indian Government implemented a flagship program NNM (National Nutrition Mission) in the first phase (2017–18) to eradicate malnutrition in this district. Before that, another flagship program, ICDS was implemented by the Indian Government during the 1990s and still in operation. Nevertheless, the prevalence of child stunting is at an alarming level. All the public health machinery from the grassroots level to a higher level, e.g., the local governing body to the State Government and Central Government are requested to monitor the implementing process and effectiveness of the programmes if the target ‘Mission 25 by 2022’ for stunting, that is still to be achieved. Further, special attention needs to be placed on the modifiable risk factors of child stunting as this study has found that preceding birth interval, low birth weight, duration of breastfeeding, mother’s education, and occupation are the associated risk factors of stunting in this district. Policy interventions should direct community health workers, e.g., ASHA (Accredited Social Health Activist), AWW (Anganwadi Worker), and ANM (Auxiliary Nurse Midwife), to encourage women as well as their male partners to increase birth interval using various family planning practices, provide extra care for preterm birth or low birth weight baby following WHO’s recommendation 2015 [63], increase educational awareness, that can help to reduce childhood stunting and thereby result in healthy childhood development. Mothers who are bidi workers and their children, as well as particular communities (AWCs), should be targeted groups as they need special attention because their children are at higher risk of being stunted. Socially most deprived ST community also needs special attention as the proportion of stunted children are highest among social groups and all other factors in this study setting. Further investigation is required as to why the children of mothers working as bidi workers are at higher risk of childhood stunting. Also, studies are needed to investigate the risk factors of stunting within the community level as well.

## Supplementary Information

**Additional file 1.**

## Data Availability

The datasets used and/or analysed during the current study are available from the corresponding author on reasonable request.

## Abbreviations

ANM: Auxiliary Nurse Midwife
ASHA: Accredited Social Health Activist
AWC: Anganwadi Centre
AWH: Anganwadi Helper
AWW: Anganwadi Worker
C2IQ: Coverage, Continuity, Intensity and Quality
CI: Confidence interval
DHS: Demographic Health Survey
ECD: Early childhood development
HAZ: Height-for-age Z-score
ICC: Intra-class correlation coefficients
ICDS: Integrated Child Development Services
IUGR: Intra-uterine growth retardation
kg: Kilogram
LMP: Last menstrual period
LR: Log-likelihood test
MICS: Multiple Indicator Cluster Survey
NFHS: National Family Health Survey
NNM: National Nutrition Mission
OBC: Other Backward Class
OR: Odds ratio
POSHAN: Prime Minister’s Overarching Scheme for Holistic Nutrition
PSUs: Primary sampling units
SC: Scheduled Caste
SD: Standard deviation
SDG: Sustainable Development Goal’s
SE: Standard error
ST: Scheduled Tribe
UNICEF: United Nations Children’s Fund
USA: United States of America
WAZ: Weight-for-age Z-score
WHO: World Health Organization
WHZ: Weight-for-height Z-score
