# Supplementary material for: Nutritional status and concomitant factors of stunting among pre-school children in Malda, India: A micro-level study using a multilevel approach
Source: BMC Public Health. 2021 Sep 16;21:1690. doi: 10.1186/s12889-021-11704-w (PMC8447797; doi:10.1186/s12889-021-11704-w)
Supplement: Supplementary file 1 — Additional file 1. [file 12889_2021_11704_MOESM1_ESM.docx]

**Supplementary file**

**ICDS**

ICDS (Integrated Child Development Services) is one of the largest and unique schemes for early childhood development in the world. It aims to break the intergenerational cycle of malnutrition, morbidity and mortality on the one hand and provide non-formal education among pre-school children on the other. The beneficiaries of this programme are children (0-6 years), lactating mothers and pregnant women. ICDS is operating through Anganwadi Centres (AWCs), which work as the first outpost for nutrition, health and early learning services at the grassroots level or the village level in rural areas and the ward level in urban areas. The AWC is operated by an Anganwadi Worker (AWW) and an Anganwadi Helper (AWH). The AWW is accountable for providing supplementary nutrition, non-formal pre-school education and health education (1–3). The number of AWC per village or ward depends upon the area and child population size, socio-economic condition and the vulnerability among children and mothers. It may vary from one to more than that per village/ward.

**Malda**

Malda is a district of West Bengal, India, located along the eastern border of the country, was selected as the study setting. Malda is one of the 315 districts of India, where NNM was implemented in the first phase during 2017-18 to eradicate malnutrition (4). According to the 2011 Census of India (5), the district had a total population of 3,988,845 that is almost equal to the national population of Georgia or the Oregon State of USA; also, it has a higher number of population than 100 countries among 233 countries which are listed in the United Nation, 2017 (6). Further, Malda ranked 58^th^ in terms of the size of the population out of the total 640 districts in India.

**Fieldwork**

The fieldwork for this survey was carried out between 01/03/2018 to 31/05/2018, spreading over a short period of about three months. The survey was conducted as a door-to-door interview. The surveyor of this fieldwork was the first author himself for this study. He is a fluent Bengali speaker and a native of Malda also. A local resident had been hired in each PSU for assisting the surveyor. The hired persons were adult, educated, and extrovert, who assisted in locating the sampled households and introducing the surveyor mainly. At the beginning of the interview, the respondents were informed well about the study and its purposes. After taking the respondents consent, survey questions were asked by the interviewer. A nine pages written questionnaire was filled up manually by the interviewer.

**Model specification**

The model here is expressed as:

$${HAZ}_{ij}={\beta'}_{Xijk}+u_{j}+e_{ij}$$

Where HAZ*_ij_* is the height-for-age Z-score for the *i*th child belongs to *j*th community. *β* is the vector of regression coefficients of *x_ijk_* that provides the coefficients of *xk* (*k* =1,….,K) for the *i*th child of the *j*th community. Where, *u_j_* represents the error term, common for all children belong to the *j*th community, and *e_ij_* denotes the deviation of *i*th child from the mean of all children belong to the *j*th community. Here, *u_j_* and *e_ij_* are assumed to be distributed normally with means equivalent to zero and variances σ^2^*_u_* and σ^2^*_e,_*, respectively (7,8). This model can be lengthened by including more number of explanatory factors. The intra-class correlation coefficients (ICC) were computed to see the association between HAZ scores of children with the same community using the formula ICC= σ^2^*_u_* / (σ^2^*_u +_* σ^2^*_e_*). Where, σ^2^*_u_* indicates the between-communities variance, and σ^2^*_e_* specifies the child or household level variance that is a fixed value of π^2^/3 for a binary outcome, mostly equal to 3.29 (9). The entire analysis was performed using STATA version 13.1 (10).

**Questionnaire**

| questionnaire for children Three-five years | | PhD topic: **Early Childhood Development and its Correlates: A Case of Malda District, West Bengal**  Rayhan Sk  Centre for the Study of Regional Development/School of Social Sciences  Jawaharlal Nehru University  New Delhi-India 110067 | | | | 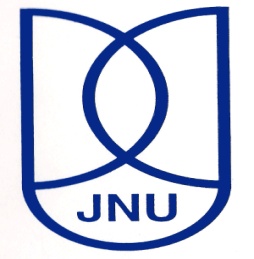 |
| --- | --- | --- | --- | --- | --- | --- |
| household information panel | | | HH | | |  |
| **HH1.** *Block number: ___ ___* | | **HH2**. *Cluster/village/ward number: ___ ___* | | | |  |
| **HH3.** *AWC name & number*  …………………………………………… 19332 _ _ _ _ _ _ _ | | **HH4**. *Household number:___ ___* | | | |  |
| **HH5**. *Day / Month / Year of the interview:*  ___ ___ /___ ___ / 2 0 1 ___ | | **HH6**. *Area:*  Urban ……………………………………………... 1  Rural..……………………………………………...2 | | | |  |
| Hello, my name is **Rayhan Sk, a PhD Scholar at the Jawaharlal Nehru University, New Delhi, India.** I am conducting a survey about the situation of children, families and households to fulfil the objectives of my PhD thesis. I would like to talk to you about these subjects. This interview usually takes about 30-35 minutes. All the information I obtain will remain strictly confidential and anonymous. Also, the findings of this study could be published online or print format. If you do not wish to answer a question or stop the interview, please let me know. May I start now? | | | | | |  |
| Yes 1  No / NOT ASKED 2 | | | | **HH7.** name & number of interviewer:  __ __ | |  |

| Women/caregiver information panel | | wc | | |
| --- | --- | --- | --- | --- |
| wc1. Name and line number of women aged 15-49 years/caregiver having at least one child aged between 36-60 months or 3-5 years | | Name  Line number………………………….……..__ __ | | |
| WC2. Father’s name:………………….. | | | | |
| *If household has more than one or more eligible women, fill up another questionnAire and put the line number of eligible women/caregiver subsequently* | | | | |
| **WC3**. Before I begin the interview, could you please bring Birth Certificate and any immunisation record for all the children?  *If anyone died after birth, please round the line number of that child and recode the duration (in days) of his/her lifespan in the* AGE *column* | child LINE no. & name; young-old  1……………………………………  2…………………………………….  3…………………………………….  4…………………………………….  5……………………………………. | SEX  M F  1 2   1. 2 2. 2 3. 2   1 2 | DATE OF BIRTH  DAY/MON/YEAR  1__ __/__ __/2 0 1 __  2__ __/__ __/2 0 1 __  3__ __/__ __/2 0 1 __  4__ __/__ __/2 0 1 __  5__ __/__ __/2 0 1 __ | AGE (completed months/years)  __ __/___  __ __/___  __ __/___  __ __/___  __ __/___ |
| **WC4.** Select one child alphabetically with age between 36-60 months from **WC3**  NAME OF CHILD IN SHORT FORM……………………………… | | LINE NUMBER OF CHILD  ………...1………………2……………….3 | | |
| **WC5**. *If mother died,* who is the primary caregiver for that child (***name***)? | | Father 1  other adult household member 2 | | |
| **WC6**. What is the **order** of birth (***name***)? | | birth order ………………………………….__ __ | | |
| **WC7.** Is (name) attending any **early childhood education** **programme** (ICDS/ECE/DAY CARE) in the current school year? | | Yes 1  NO 2 | | |
| **WC8**. *If yes,* what is the **type of the early childhood education** **programme**? | | ICDS/Anganwadi 1  NGO 2  Private pre-SCHOOL..………………………3  Other (specify)………………………….………6 | | |
| **WC9**. How long did (he/she) attend ECE? | | duration in months…………….._____ | | |
| **WC10**. Is there any **home tuition** for that child (name)? | | Yes 1  No …2 | | |
| **WOMAN’S/CAREGIVER BACKGROUND WB** | | | | |
| **WB1**. How **old** are you? | | Age (in completed years) __ __ | | |
| **WB2**. How many **children** do you have? | | total number of Children __ __ | | |
| **WB3**. How many **children live** with you? | | Children at home __ __ | | |
| **WB4**. Was (***name***) delivered by **caesarean section**? | | Yes 1  No 2 | | |
| **WB5**. Where was the child (***name***) **delivered**? | | place of delivery  Home……………………………………………..1  public hospitals…………………………….2  Private hospitals…………………………..3  other place…………………………………..4 | | |
| **WB6.** What was the number of **antenatal care checkups** (ANC) of child **(*name*)**? | | None…………………………………………….0  number of anc visits………………..__ __ | | |
| **WB7**. When (***name***) was born, was (he/she) very large, larger than average, average, smaller than average, or very small? | | Very large 1  Larger than average 2  Average 3  Smaller than average 4  Very small 5  DK 8 | | |
| **WB8.** What was the date of **last menstrual period** (LMP) for the child (name)? *recode from immunisation card* | | DATE OF LMP DAY/MON/YEAR  ………………………… _ _/ _ _ / 201_ | | |
| **WB9**. Was (***name***) **weighed** at birth? | | Yes 1  No 2  DK 8 | | |
| **WB10**. *If weighted*, how much did (***name***) **weigh**? | | From card/recall  **(grams)** __ __ __ __  DK 99 | | |
| **WB11**. What is the highest **level of school** you have attended? | | None ……………………………………………..0  Primary …………………………………………1  Lower Secondary …………………………..2  Upper Secondary ……………………………3  Higher…………………………………………..4 | | |
| **WB12**. What is your **occupation**? | | Housewive……………………………………...1  Agriculture…………………………………...2  manual labour………………………………3  biri worker……………………………………4  govt. service………..………………………...5  priv. service…………………………………...6  *If* other *specify*___________________________7 | | |
| **WB13**. How much time do you give to your child (name) **for daily care**? | | tIME IN HOURS…………………………___ ___ | | |
| **WB14**. How much time do you give to your child (name) **for study purpose** (reading/writing/drawing/singing etc.? | | tIME IN HOURS…………………………___ ___ | | |
| **WB15**. What is your **marital status** now? | | Widowed 1  Divorced 2  Separated ……………………………………...3 | | |
| **WB16**. Besides yourself, does your (husband/partner) have any other wives? | | Yes 1  No 2 | | |
| **WB17**. *If yes,* how many other wives does he have? | | Number __ __  DK .99 | | |
| **WB18**. Does your **husband live** with you? | | yes…………………………………………………1  no..…………………………………………………2  died………………………………………………...3 | | |
| **WB19**. *If no*, where is he staying? | | workplace………………………………………1  Don’t know……………………………………...8 | | |
| **WB20**. If your husband is staying in the workplace or doing work at far from you, how long did your husband stay with you during last year? | | duration in months……………..____ ____ | | |
| **WB21**. What is the highest **level of the school** of your **husband** have attended? | | None ……………………………………………..0  Primary …………………………………………1  Lower Secondary …………………………..2  Upper Secondary ……………………………3  Higher…………………………………………..4 | | |
| **WB22**. What is your **husband occupation**? | | none……….……………………………………...1  Agriculture…………………………………...2  manual labour………………………………3  biri worker……………………………………4  govt. service…….….………………………...5  priv. service…………………………………...6  *If* other *specify*___________________________7 | | |
| **WB23**. How much time does your **husband give** to your child (name) for **daily care**? | | tIME IN HOURS…………………………_____ | | |
| **WB24**. How much time does your **husband give** to your child (name) for **study purpose** (reading/writing/drawing/singing etc.? | | tIME IN HOURS…………………………_____ | | |
| **WB25**. Who does give the **maximum time** to the child (name)? | | MOTHER..….……………………………………...1  Father…………………………………………...2  Grand mother..………………………………3  Grand father…………………………………4  other adult member.……………………...5 | | |
| **WB26**. Does your husband have any **drinking (alcoholic**) habit? | | Yes 1  No 2  don’t want to say………………………….3 | | |
| **WB27.** *If yes*, what is the **nature** of your husband drinking (alcoholic) habit? | | daily……………………………………………1  weekly.………………………………………..2  monthly……..………………………………..3  yearly…………………………………………4 | | |
| **WB28**. Have you experienced any **domestic violence** (beaten up/verbal abuse) by your husband or other member of the household during last three months? | | Yes 1  No 2  don’t want to say………………………….3 | | |
| **WB29**. *If yes,* how many times did you experience any violence during the last month? | | frequency of the incidence……..______ | | |
| **WB30**. What is the **relation** to the household head? | | self………………………………………………1  husband………………………………………..2  mother inlaw………………………………..3  father inlaw…………………………………4  other member of household……………..5 | | |
| **WB31**. What is your **family type**? | | single……………………………………………1  joint………………………………………………2 | | |
| **WB32**. Do you own a **mobile phone**? | | Yes 1  No 2 | | |
| **WB33**. *If yes,* is it a **smart phone**? | | No/normal 0  yes 1 | | |
| **WB34.** *If it is a smart phone,* does it has an **internet connection**? | | Yes 1  No 2 | | |
| **WB35.** *If it is a smart phone,* is the child (name) **accessing the smart mobile phone** and playing a game or watching the video? | | Yes 1  No 2 | | |
| **WB36**. In the past 12 months, have you personally felt discriminated against or harassed on the basis of the following grounds?  [A] Caste?  [E] Religion?  [F] Disability? | | Yes No DK  cast 1 2 8  Religion 1 2 8  Disability 1 2 8 | | |
| **WB37**. Are you suffering from any **chronical or other diseases** which spanned over three months or more during last one year? | | Yes 1  No 2 | | |
| **WB38**. I would like to ask you some simple questions on **happiness.**  *Are you (pronounce the happiness levels)?* | | Very happy 1  Somewhat happy 2  Neither happy nor unhappy 3  Somewhat unhappy 4  Very unhappy……………………….................5 | | |
| **EARLY CHILDHOOD DEVELOPMENT EC** | | | | |
| **EC1**. How many **children’s books** or picture books do you have for (**name**)? | | Number of children’s books _ _ | | |
| **EC2**. I am interested in learning about the things that (**name**) plays with when (he/she) is at home | | Does (he/she) play with: | | |
| [A] **Homemade** toys, such as dolls, cars, or other toys made at home? | | Yes 1  No 2  DK………………………………………………….8 | | |
| [B] Toys from a **shop or manufactured** toys? | | Yes 1  No 2  DK………………………………………………….8 | | |
| [C] Household objects, such as bowls or pots, or objects found outside, such as sticks, rocks, animal shells or leaves? | | Yes 1  No 2  DK………………………………………………….8 | | |
| **EC3**. Sometimes adults taking care of children have to leave the house to go shopping, wash clothes, or for other reasons and have to leave young children. | | On how many days in the past week was (**name**): | | |
| [A] **Left alone** for more than an hour? | | Number of days left alone for  more than an hour ___  No………………………………………………….0  DK………………………………………………….99 | | |
| [B] **Left in the care of another child**, that is, someone less than 10 years old, for more than an hour? | | Number of days left with another child for more Than an hour ___  No………………………………………………….0  DK………………………………………………….99 | | |
| **EC4**. **In the past 3 days,** did you or any household member age 15 or over engage in any of the following activities with (name): | | If ‘Yes’, ask: Who engaged in this activity with (name)? | | |
| [A] **Read books** or looked at picture  books with (**name**)? | | mother………………………………………A  father….……………………………………B  other…………………………………………X  no one…………………………….…………Y | | |
| [B] **Told stories** to (**name**)? | | mother………………………………………A  father….……………………………………B  other…………………………………………X  no one…………………………….…………Y | | |
| [C] **Sang songs** to or with (**name**),  including lullabies? | | mother………………………………………A  father….……………………………………B  other…………………………………………X  no one…………………………….…………Y | | |
| [D] Took (**name**) **outside** the home? | | mother………………………………………A  father….……………………………………B  other…………………………………………X  no one…………………………….…………Y | | |
| [E] **Played** with (**name**)? | | mother………………………………………A  father….……………………………………B  other…………………………………………X  no one…………………………….…………Y | | |
| [F] **Named, counted**, or drew things  for or with (**name**)? | | mother………………………………………A  father….……………………………………B  other…………………………………………X  no one…………………………….…………Y | | |
| **EC5**. Can (**name**) **identify** or name at least ten letters of the alphabet? | | Yes 1  No 2  DK……………………………………………………8 | | |
| **EC6**. Can (**name**) **read** at least four simple, popular words? | | Yes 1  No 2  DK 8 | | |
| **EC7**. Does (**name**) know the **name and recognise** the symbol of all numbers from 1 to 10? | | Yes 1  No 2  DK 8 | | |
| **EC8**. Can (**name**) **count the number** from 1 to 10 or walk 10 steps with counting? | | Yes 1  No 2  DK 8 | | |
| **EC9**. Can (**name**) **pick up a small** **object** with two fingers, like a stick or a rock from the ground? | | Yes 1  No 2  DK 8 | | |
| **EC10**. Is (**name**) sometimes **too sick** to play? | | Yes 1  No 2  DK 8 | | |
| **EC11**. Does (**name**) follow **simple directions** on how to do something correctly? | | Yes 1  No 2  DK 8 | | |
| **EC12**. When given something to do, is (**name**) able to do it **independently**? | | Yes 1  No 2  DK 8 | | |
| **EC13**. Does *(***name**) **get along well** with other children? | | Yes 1  No 2  DK 8 | | |
| **EC14**. Does (**name**) **kick, bite, or hit** other children or adults? | | Yes 1  No 2  DK 8 | | |
| **EC15**. Does (**name**) get **distracted easily**? | | Yes 1  No 2  DK 8 | | |
| **breastfeeding and dietary intake Bd** | | | | |
| **BD1.** Has (name) ever been **breastfed?** | | Yes 1  No 2  DK 8 | | |
| **BD2.** *If yes,* how long did (he/she) **breastfed**? | | Duration in months…. _____  DK 99 | | |
| **CARE OF ILLNESS CA** | | | | |
| **CA1.** In the last two weeks, has (**name**) had **diarrhoea**? | | Yes 1  No 2  DK 8 | | |
| **CA2**. *If yes,* did you seek any advice or **treatment** for the diarrhoea from any source? | | Yes 1  No 2  DK 8 | | |
| **CA3**. At any time in the last two weeks, has (**name**) been ill with a **fever**? | | Yes 1  No 2  DK 8 | | |
| **CA4**. At any time in the last two weeks, has (**name**) had an illness with a **cough?** | | Yes 1  No 2  DK 8 | | |
| **CA5**. At any time in the last two weeks, has (***name***) had fast, short, rapid breaths or **difficulty breathing**? | | Yes 1  No 2  DK 8 | | |
| **CA6**. Was the fast or difficult breathing due to a problem in the chest or a blocked or runny nose? | | Problem in chest only 1  Blocked or runny nose only 2  Both 3  Other (specify) 6  DK 8 | | |
| **CA7**. Did you seek any advice or **treatment** for the illness (CA3, 4, 5, 6 from any source? | | Yes 1  No 2  DK 8 | | |
| **CA8.** Is the child (name) suffered from any **major disease or injury** during his/her lifetime? | | Yes 1  No 2 | | |
| **CA9.** Did you or any other household member physically punish the child **(name)** in the last week? | | Yes 1  No 2 | | |
| **CA10.** *If yes,* how many times the child was punished in last week? | | frequency of phy. punishment………__ __ | | |
| **ANTHROPOMETRY AN** | | | | |
| **AN1**. *Record the result of* ***weigh****t measurement as read out by the Measurer:* | | Kilograms (kg) ___ ___ **.** ___  Child not present 96  Child refused 99 | | |
| **AN2**. *Record the result of* ***height*** *measurement as read out by the Measurer:* | | Length / Height (cm) ___ ___ ___ . ___  Child refused 99 | | |
| **Household characteristics HC** | | | | |
| *If the household number of the respondent is same as interviewed earlier respondent, then refer the previous household number only and do not ask further questions* | | same as household no:……….…..___ ___ | | |
| **HC1.** What is the **total number** of the household member? | | number of household member…….___ ___ | | |
| **HC2.** What is the **religion** of (***name of the head of the household***)? | | Hindu…………………………………….1  Muslim…………………………………..2  Christian……………………………….3  Other religion(specify) ……6  No religion…………………………….7 | | |
| **HC3**. What is the **mother tongue**/native language of (***name of the head of the household***)? | | Bangla…………………………………1  Hindi…………………………………….2  Other language (specify)……………………..6 | | |
| **HC4**. To what **Caste group** does (***name of the head of the household***) belong? | | General………………………………1  OBC ……………………………………..2  Scheduled Caste ….………………3  Scheduled Tribe …………………….4 | | |
| **HC5.** How many **rooms** do members of this household usually use for **sleeping**? | | Number of rooms………………__ __ | | |
| **HC6**. *Main material of the* ***dwelling floor.***  *Record observation.* | | Earth / Sand 1  Wood planks 2  Ceramic tiles 3  Cement 4  Carpet 5 | | |
| **HC7**. *Main material of* ***the roof****.*  *Record observation.* | | Thatch / grass 1  Plastic 2  Metal / Tin 3  Wood 4  MUD tiles 5  Cement 6 | | |
| **HC8**. *Main material of the* ***exterior walls.***  *Record observation.* | | Grass/Cane / Palm / 1  MUD 2  Bamboo with mud 3  Stone with mud 4  Brick with mud 5  Brick with Cement 6  Cement blocks 7 | | |
| **HC9.** Does your household **have electricity**? | | Yes 1  No………………………………………………..2 | | |
| **HC10**. Does your household have:  [A] A television?  [B] A refrigerator?  [C] A washing machine? | | Yes No  Television 1 2  Refrigerator 1 2  WASHING MACHINE………………..1 2 | | |
| **HC11**. Does any member of your household own:  [B] A bicycle?  [C] A motorcycle or scooter?  [D] An animal-drawn cart?  [E] A car, truck or van? | | Yes No  Bicycle 1 2  Motorcycle / Scooter 1 2  Animal-drawn cart 1 2  Car / Truck / Van 1 2 | | |
| **HC12**. Does any member of your household **have a computer or a tablet?** | | Yes 1  No 2 | | |
| **HC13**. Does your household have access to **internet at home**? | | Yes 1  No 2 | | |
| **HC14**. Do you or someone living in this household **own this dwelling**? | | Own 1  Rent 2  Other (*specify)* 6 | | |
| **HC15**. Does any member of this household **own any land** that can be used for agriculture? | | Yes 1  No 2 | | |
| **HC16**. How many of agricultural land do members of this household own? *If less than 1, record ’00’.* | | BIGHAS ___ ___  DK 99.2 | | |
| **HC17**. Does this household **own any livestock**, herds, other farm animals, or poultry? | | Yes 1  No 2 | | |
| **HC18**. How many of the following animals does this household have?  [A] Milk cows or bulls?  [B] Other cattle?  [C] Horses?  [D] Goats?  [E] Sheep?  [F] Chickens?  [G] Pigs? | | milk cows or bulls ___ ___  Other cattle ___ ___  Horses ___ ___  Goats ___ ___  Sheep ___ ___  Chickens ___ ___  Pigs ___ ___ | | |
| **HC19**. Does any member of this household **have a bank account**? | | Yes 1  No 2 | | |
| **HC20**. What **type of fuel** is used **for cooking**? | | Solar air heater 1  electricity 2  LPG/cooking gas 3  Biogas 4  Kerosene / paraffin 5  Coal / Lignite 6  Charcoal 7  Wood 8  Crop residue/grass/Stra…………………..9  Animal dung / waste 10  Garbage / Plastic 11  sawdust 12  Other (specify) 6 | | |
| **HC21**. Is the cooking usually done in the house, in a separate building, or outdoors? | | **In main house**  no Separate room 1  in a separate room 2  in sepa. building/outdoors. 3  Other (specify) 6 | | |
| **HC22**. At night, what does your household mainly use to **light the household?** | | Electricity 1  bioGas lamp 2  Kerosene or paraffin lamp 3  Other (specify) 6 | | |
| **HC23**. What is the main source of **drinking water** used by members of your household? | | Piped into dwelling 11  Public tap / standpipe 14  Tube Well / Borehole 21  Protected well 31  Unprotected well 32  water kiosk 72  Surface water (river, lake, pond) 81  Bottled water 91  Other (specify) 6 | | |
| **HC24**. Where is that **water source located**? | | In own dwelling 1  In own yard / plot 2  Elsewhere 3 | | |
| **HC25**.What kind of **toilet facility** do members of your household usually use?    If not possible to determine, ask permission to observe the facility. | | **Flush / Pour flush**  Flush to piped sewer system 11  Flush to septic tank 12  Flush to pit latrine 13  Flush to open drain 14  Flush to DK where 18  **Pit latrine**  Ventilated Improved Pit  latrine 21  Pit latrine with slab 22  Pit latrine without slab 23  Hanging toilet 51  No facility / Bush / Field 95  Other (specify) 6 | | |
| **HC26**. Do you have any **soap or detergent or liquid** soap in your house for washing hands? | | Yes 1  No 2 | | |
| **HC27.** What is your or household **annual/per year income**? | | annual Income (rs)……………___________  don’t want to say…………………………..99 | | |

**References**

1. Rao N, Kaul V. India’s integrated child development services scheme: challenges for scaling up. Child Care Health Dev [Internet]. 2018 Jan;44(1):31–40. Available from: http://doi.wiley.com/10.1111/cch.12531

2. Narayan J, John D, Ramadas N. Malnutrition in India: status and government initiatives. J Public Health Policy [Internet]. 2019 Mar 23;40(1):126–41. Available from: https://doi.org/10.1057/s41271-018-0149-5

3. Ministry of Women and Child Development. The government’s efforts to fight malnutrition [Internet]. 2013. Available from: http://iap.healthphone.org/pdf/the-governments-efforts-to-fight-malnutrition.pdf

4. Ministry of Women and Child Development. National Nutrition Mission (NNM): States/UTs-wise Districts covered in Phase I (Year 2017-18) [Internet]. New Delhi; 2018. Available from: https://icds-wcd.nic.in/nnm/NNM-Web-Contents/RIGHT-MENU/NNM-States-Districts/NNM-Districts-315-Phase-I.pdf

5. Registrar General of India. Primary Census Abstrat. New Delhi; 2011.

6. United Nations, Department of Economic and Social Affairs PD. World Population Prospects The 2017 Revision [Internet]. New York; 2017. Available from: https://esa.un.org/unpd/wpp/publications/files/wpp2017_keyfindings.pdf

7. Kanjilal B, Mazumdar P, Mukherjee M, Rahman MH. Nutritional status of children in India: household socio-economic condition as the contextual determinant. Int J Equity Health [Internet]. 2010;9(1):19. Available from: http://equityhealthj.biomedcentral.com/articles/10.1186/1475-9276-9-19

8. Griffiths P, Matthews Z, Hinde A. Gender, family, and the nutritional status of children in three culturally contrasting states of India. Soc Sci Med [Internet]. 2002 Sep;55(5):775–90. Available from: https://linkinghub.elsevier.com/retrieve/pii/S0277953601002027

9. Goldstein H, Browne W, Rasbash J. Partitioning Variation in Multilevel Models. Underst Stat. 2002;1(4):223–31.

10. StataCorp. Stata Statistical Software: Release 13. Stata: Release 13. College Station, TX; 2013.
